# Skin-bacteria communication: Involvement of the neurohormone Calcitonin Gene Related Peptide (CGRP) in the regulation of *Staphylococcus epidermidis* virulence

**DOI:** 10.1038/srep35379

**Published:** 2016-10-14

**Authors:** Awa R. N’Diaye, Camille Leclerc, Takfarinas Kentache, Julie Hardouin, Cecile Duclairoir Poc, Yoan Konto-Ghiorghi, Sylvie Chevalier, Olivier Lesouhaitier, Marc G. J. Feuilloley

**Affiliations:** 1 Laboratory of Microbiology Signals and Microenvironnement, LMSM, EA 4312, Normandie Université, Evreux, France; 2 Laboratory of Polymers, Biopolymers and Surfaces, CNRS UMR 6270, Normandie Université, Mont-Saint-Aignan, France

## Abstract

*Staphylococci* can sense Substance P (SP) in skin, but this molecule is generally released by nerve terminals along with another neuropeptide, Calcitonin Gene Related Peptide (CGRP). In this study, we investigated the effects of αCGRP on *Staphylococci.* CGRP induced a strong stimulation of *Staphylococcus epidermidis* virulence with a low threshold (<10^−12 ^M) whereas *Staphylococcus aureus* was insensitive to CGRP. We observed that CGRP-treated *S. epidermidis* induced interleukin 8 release by keratinocytes. This effect was associated with an increase in cathelicidin LL37 secretion. *S. epidermidis* displayed no change in virulence factors secretion but showed marked differences in surface properties. After exposure to CGRP, the adherence of *S. epidermidis* to keratinocytes increased, whereas its internalization and biofilm formation activity were reduced. These effects were correlated with an increase in surface hydrophobicity. The DnaK chaperone was identified as the *S. epidermidis* CGRP-binding protein. We further showed that the effects of CGRP were blocked by gadolinium chloride (GdCl_3_), an inhibitor of MscL mechanosensitive channels. In addition, GdCl_3_ inhibited the membrane translocation of EfTu, the Substance P sensor. This work reveals that through interaction with specific sensors *S. epidermidis* integrates different skin signals and consequently adapts its virulence.

Skin is the major neuroendocrine organ of the human body^1^ and harbors a large and highly diverse microbiota^2^. Skin bacteria are continuously exposed to host molecules that diffuse through to the upper layers of the epidermis and are present in sweat^3^. Many of these molecules are released by afferent nerve terminals and show important local variations in their concentration in response to stress, inflammation or even pain^1^,^4^. We have previously shown that skin bacteria of different phyla, including gram-positive species such as *Bacillus cereus*^5^, *Staphylococcus aureus*^5^,^6^ and *Staphylococcus epidermidis*^5^,^6^, and gram-negative bacteria such as *Pseudomonas fluorescens*^7^ can detect Substance P (SP), the principle skin neuropeptide. This molecule between nano- and micro-molar concentrations acts on bacteria and leads to a general increase in virulence. There is now ample evidence that bacteria can detect a large range of neurohormones^8^. In skin nerve terminals, SP is frequently co-localized and co-secreted with Calcitonin Gene Related Peptide (CGRP)^9^ another neuropeptide abundantly expressed in the skin^10^.

CGRP is a 37 amino acids peptide that belongs to the calcitonin superfamily of hormones including calcitonin, amylin, and adrenomedullin. Two isoforms of this peptide—α-CGRP (or CGRP I) and β-CGRP (or CGRP II)—are produced from the same gene by alternative splicing, but α-CGRP is the major form expressed in sensory skin fibers^1^. CGRP is one of the most potent vasodilatory neuromediators and it potentiates the effects of Substance P on vascular permeability and edema formation in skin^1^. In addition, CGRP exerts trophic effects on endothelial cells and melanocytes. Regarding the effects of this family of peptides on bacteria, a direct antimicrobial effect of CGRP has been described toward *Escherichia coli* and *Pseudomonas aeruginosa*^11^ and is explained by the structural similarities between CGRP and antimicrobial peptides. However the antimicrobial spectrum of CGRP is limited and it has been shown to have no effect on *Staphylococcus aureus* viability^11^. The ability of bacteria to detect and respond to neuropeptides through specific sensors and mechanisms was recognized only at the end of the 20^th^ century and has diffused slowly in the scientific community under the name “Microbial Endocrinology”^12^. Many peptides initially identified for entirely unrelated functions that have secondary antimicrobial activity at non-physiological concentrations, such as natriuretic peptides, have been found to be able to modulate bacterial adhesion, biofilm formation activity and virulence through specific interaction with sensor proteins^13^. However, to date the physiological effects of CGRP on bacteria and its potential detection by bacterial sensory proteins have not been investigated.

In the present report, we studied the effect of αCGRP on *S. aureus* and *S. epidermidis,* two of the major bacterial species constituting the skin microbiota. Strains isolated from normal human skin were used to avoid the bias that are potentially encountered when library reference strains are used. The effect of αCGRP on *S. aureus* and *S. epidermidis* virulence was investigated in cultured keratinocytes and reconstructed human epidermis to more closely simulate normal skin conditions. Although they are of the same genus, these bacteria had completely different sensitivities to CGRP. The CGRP sensor was identified in *S. epidermidis,* and the mechanism of action of the peptide was partly elucidated. A schema was then proposed to explain how *S. epidermidis* can sense and integrate different host signals and adapt its virulence in response to a cutaneous environment.

## Results

### CGRP stimulates the cytotoxicity of *Staphylococcus epidermidis* on cultured keratinocytes and reconstructed human epidermis

**Figure 1:**
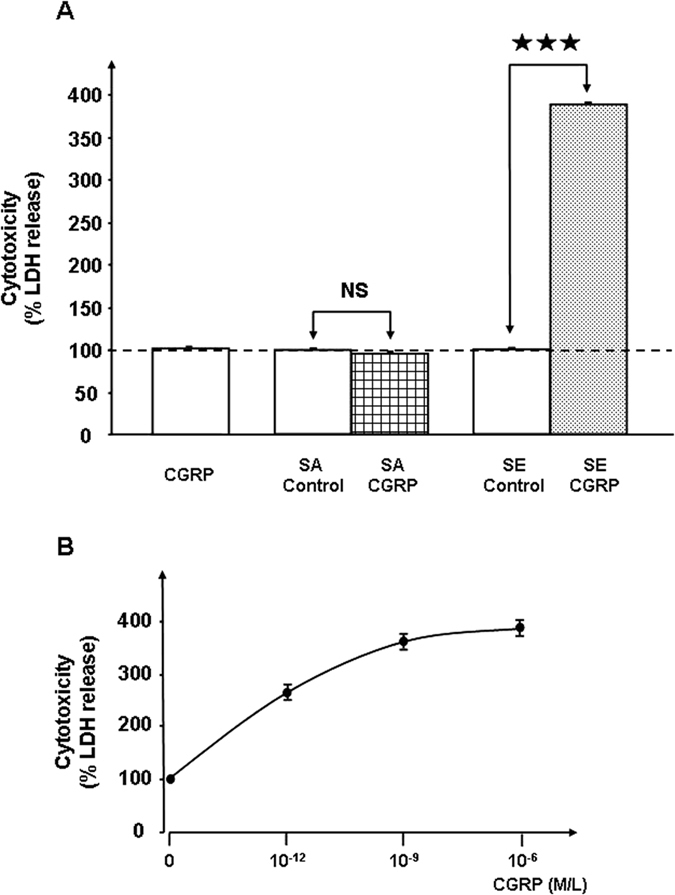
Effect of CGRP on the cytotoxic activities of *S. aureus* and *S. epidermidis*. (**A**) Comparative effect of CGRP 10^−6 ^M on the cytotoxicity of *S. aureus* MFP03 (SA) and *S. epidermidis* MFP04 (SE) toward HaCaT keratinocytes. The dotted line indicates the basal cytotoxicity level (100%) in control HaCaT cell cultures. (**B**) Dose response curve of the effect of CGRP on *S. epidermidis* MFP04 cytotoxicity (NS = not significantly different; ^★★★^*p* < 0.001). The results are representative of three independent experiments.

**Figure 2:**
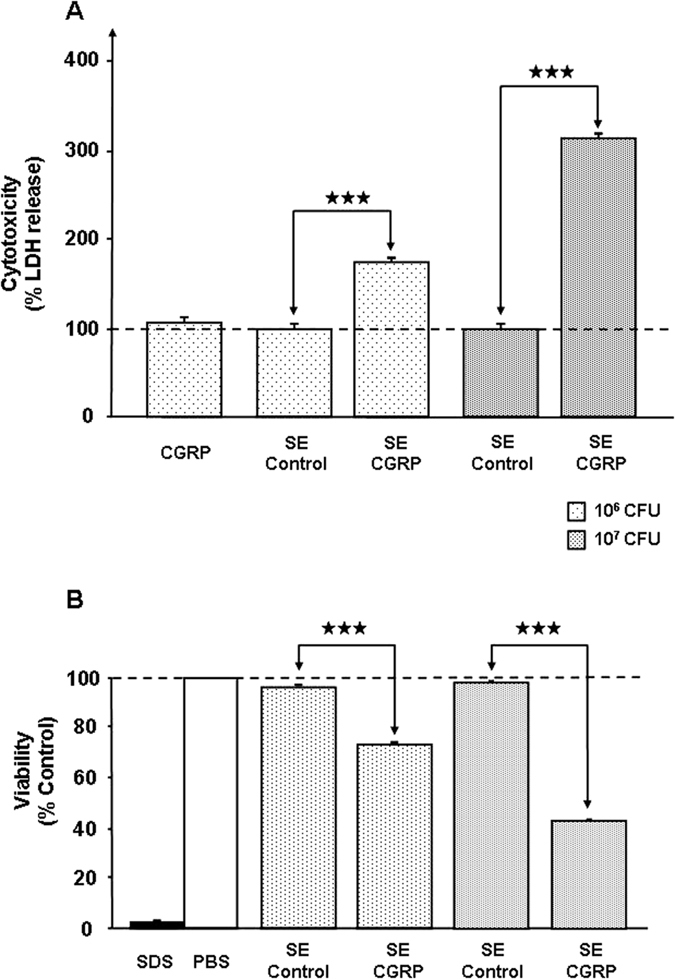
Effects of CGRP (10^−6 ^M) on the virulence of *S. epidermidis* toward RHE. The effect of CGRP on the virulence of *S. epidermidis* MFP04 (SE) was studied on RHE (SkinEthic™) by using bacterial cultures at 10^6^ and 10^7^ CFU/mL and two different techniques: assay of lactate dehydrogenase (LDH) release by dying cells (**A**) and measure of mitochondrial respiratory activity (viability) by MTT assays (**B**). The dotted lines indicate the basal cytotoxicity levels (100%) measured in RHE cultures using the two techniques. (^★★★^*p* < 0.001). The results are representative of three independent experiments.

**Figure 3:**
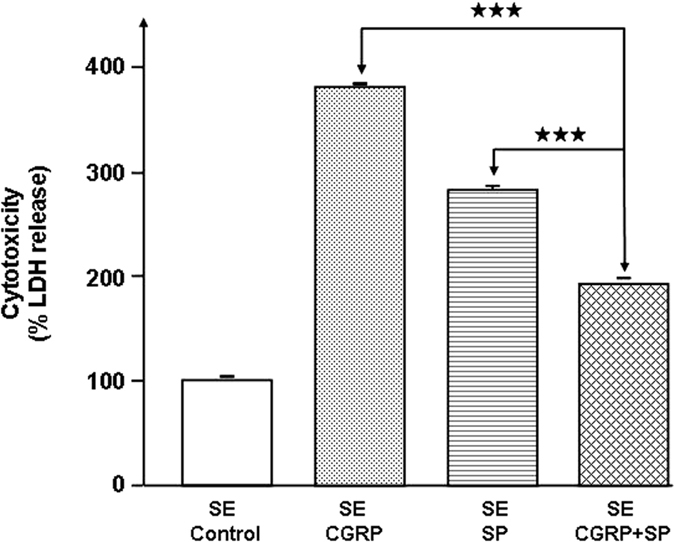
Combined effects of CGRP and Substance P on the cytotoxic activity of *S. epidermidi*s. The comparative effects of CGRP and Substance P (SP) alone or in association (10^-6 ^M each) on the cytotoxic activity of *S. epidermidis* (SE) were studied in HaCaT cells. (^★★★^*p* < 0.001). The results are representative of three independent experiments.

Preliminary experiments showed that exposure of *S. aureus* (MFP03) or *S. epidermidis* (MFP04) to CGRP (10^−6 ^M) over the entire growth phase did not modify the growth kinetics of these bacteria. The effect of CGRP on bacterial cytotoxicity was evaluated on HaCaT keratinocytes by measurement of lactate dehydrogenase (LDH), a stable cytosolic enzyme released during cell lysis^14^. CGRP (10^−6 ^M) did not affect the cytotoxicity of *S. aureus* toward HaCaT cells (Fig. 1A). In contrast, when *S. epidermidis* was previously treated with CGRP (10^−6 ^M), the bacterium showed a strong increase in cytotoxicity (+388 ± 8%, *p* < 0.001). As a control, CGRP (10^−6 ^M) was administered alone and had no effect on HaCaT keratinocytes viability. Because the bacteria were rinsed to remove any traces of free peptide before being layered on keratinocytes, this result can be explained only by a direct action of CGRP on *S. epidermidis*. Lower concentrations of CGRP were tested on *S. epidermidis* to investigate the dose-response relationship. This study showed that the threshold of the CGRP effect on *S. epidermidis* was remarkably low (<10^−12^ M) (Fig. 1B). These results were confirmed by using reconstructed human epidermidis (RHE) exposed to control or CGRP-treated *S. epidermidis* (10^6^ and 10^7^ CFU) and two different techniques to exclude any artefacts. In the first case, the cytotoxicity of bacteria was measured through assays of LDH release, as previously described. As observed with HaCaT cells, CGRP (10^−6 ^M) alone had no effect on RHE viability. When RHE was exposed to 10^6^ CFU *S. epidermidis*, the cytotoxicity of CGRP-treated bacteria increased (+173 ± 3%, *p* < 0.001), whereas that of the control did not (Fig. 2A). Using a ten-fold higher inoculate (10^7^ CFU) did not change the cytotoxicity of the control bacteria but induced a higher increase in the cytotoxicity of CGRP-treated *S. epidermidis* (+315 ± 7%, *p* < 0.001). In a second series of experiments, the virulence of *S. epidermidis* on RHE was measured by using MTT assays, which reflect mitochondrial respiratory activity and therefore cell viability^15^. A significant decrease in RHE viability was observed in the presence of 10^6^ and 10^7^ CFU *S. epidermidis* previously exposed to CGRP (−23 ± 2% and −56 ± 4%, *p* < 0.001, respectively) (Fig. 2B). Because CGRP stimulates the virulence of *S. epidermidis,* as previously observed using Substance P^6^ and because the two peptides are generally released by the same nerve fibers^9^, *S. epidermidis* was exposed simultaneously to Substance P and CGRP. Used alone and at the same concentration, the effect of CGRP on *S. epidermidis* cytotoxicity was stronger than that of Substance P (Fig. 3). However, when bacteria were pre-treated with both peptides simultaneously (10^−6 ^M each), the cytototoxicity of *S. epidermidis* was reduced relative to that of CGRP-treated bacteria (−50 ± 8%, *p* < 0.001) and Substance P-treated bacteria (−32 ± 12%, *p* < 0.001) (Fig. 3).

### CGRP-treated *Staphylococcus epidermidis* stimulates Interleukin 8 and cathelicidin secretion by keratinocytes

**Figure 4:**
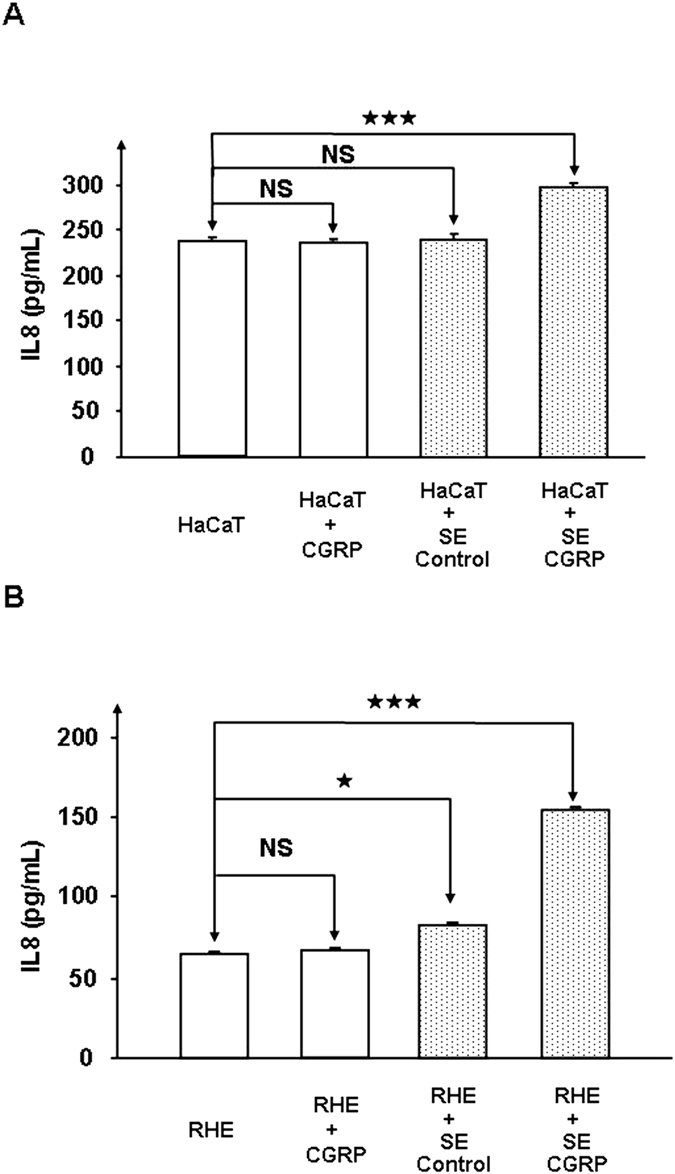
Effects of CGRP (10−6 M) on *S. epidermidis*-induced expression of IL8 in cultured HaCaT keratinocytes (**A**) and RHE (**B**). IL8 was assayed in the culture medium of HaCaT cells and RHE (SkinEthic™) exposed to control or CGRP-treated bacteria. (NS = not significantly different; ^★^*p* < 0.05; ^★★★^*p* < 0.001). The results are representative of three independent experiments.

**Figure 5:**
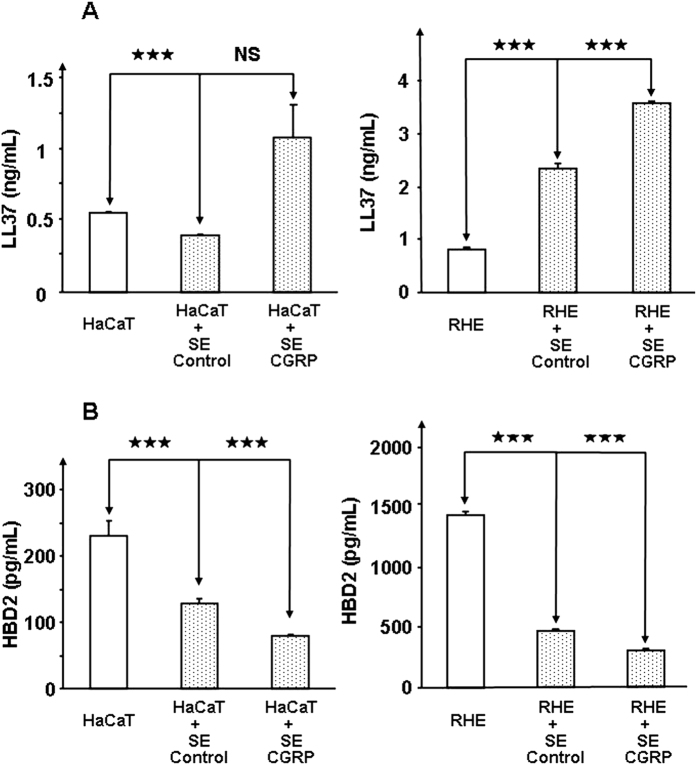
Effects of CGRP (10^−6 ^M) on *S. epidermidis-*induced expression of antimicrobial peptides in cultured HaCaT keratinocytes (**A**) and RHE (**B**). Cathelicidin LL37 and β-defensin 2 (HBD2) were assayed in the medium of HaCaT cells and RHE (SkinEthic™) exposed to control or CGRP-treated bacteria. (NS = not significantly different; ^★★★^*p* < 0.001). The results are representative of three independent experiments.

Because keratinocytes develop an efficient innate immune response to bacteria through activation of pro-inflammatory and antimicrobial peptides^16^, the production of interleukin 8 (IL8), cathelicidin (LL37) and β-defensin 2 (HBD2) by HaCaT cells and RHE was measured after exposure to control or CGRP-treated *S. epidermidis.* The basal level of IL8 secretion by HaCaT keratinocytes was not changed when the cells were exposed to CGRP (10^−6 ^M) alone or to control bacteria (Fig. 4A). In contrast, CGRP-treated *S. epidermidis* triggered a significant increase in IL8 secretion (+24 ± 3%, *p* < 0.001). Similar results were obtained using RHE, although the range of the response was amplified (Fig. 4B). By itself, the cutaneous strain of *S. epidermidis* MFP04 induced a significant increase in IL8 production by RHE (+28 ± 5%, *p* < 0.5), but when the bacteria were previously exposed to CGRP, a much more substantial stimulation of IL8 secretion was observed (+138 ± 8%, *p* < 0.001). HaCaT cells exposed to untreated bacteria showed a decrease in LL37 secretion (−28 ± 4%, *p* < 0.001) whereas an insignificant increase in LL37 was observed when they were exposed to CGRP-treated *S. epidermidis* (Fig. 5A). The response of RHE was more acute because control and CGRP-treated *S. epidermidis* induced a stimulation of LL37 (+208 ± 12% *p* < 0.001 and +343 ± 8% *p* < 0.001, respectively) (Fig. 5A). However, LL37 production was significantly higher when the cells were exposed to CGRP-treated bacteria relative to the control bacteria (*p* < 0.001). In parallel, the secretion of HBD2 by both cultured keratinocytes and RHE was significantly reduced when they were exposed to control *S. epidermidis* (−44 ± 12% and −67 ± 2%, *p* < 0.001, respectively). The decrease in HBD2 production was more important than when keratinocytes and RHE were exposed to CGRP-treated bacteria (−67 ± 11% and −79 ± 2% (Fig. 5B)).

### The effects of CGRP on *Staphylococcus epidermidis* cannot be explained by over-expression of virulence proteins

**Figure 6:**
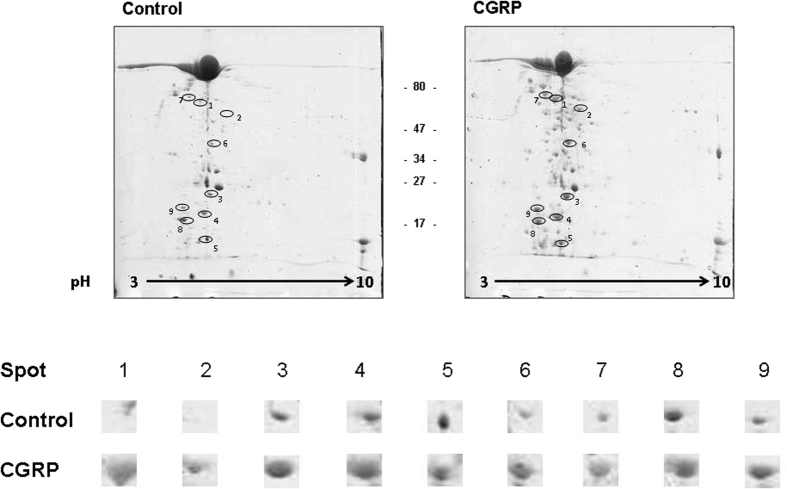
Bidimensional electrophoresis analysis of secreted *S. epidermidis* proteins in control conditions and after a 2 h treatment with CGRP (10^−6 ^M). Nine spots were identified, corresponding to proteins significantly up-regulated after exposure of the bacteria to CGRP. Proteins corresponding to these spots are presented in Table 1. The results are representative of three independent experiments.

To investigate the origin of the increase in *S. epidermidis* virulence induced by CGRP, proteins were extracted from the secretomes of control and CGRP-treated bacteria, analyzed by 2D-gel electrophoresis and identified by Matrix-Assisted Laser Desorption Ionization Time-of-Flight mass spectrometry (MALDI-TOF/TOF). Image analysis of 3 replicates of 2D gels allowed the detection of nine major proteins over-expressed in the CGRP treated *S. epidermidis* condition compared with the control condition (Fig. 6). Interestingly, among these proteins, superoxidase dismutase (Spot 3), alkaline shock protein 23 (Spot 5), DnaK protein (Spot 7) and peroxidoxin (Spot 9) are required for bacterial adaptation to stress (Table 1). Other identified proteins, including dihydrolipoyl dehydrogenase (Spot 1), inosine-monophosphate dehydrogenase (Spot 2) and citrate synthase (Spot 6), are involved in intermediate metabolism. Only one of the proteins over-expressed in CGRP-treated *S. epidermidis*, immunodominant surface antigen B (IsaB) (Spot 3) and its precursor (Spot 8), is considered to be a virulence factor because of its antigenic activity^17^. In addition, tandem mass spectrometry analysis identified of a core of 170 proteins reproducibly found in the secretome of control and CGRP-treated *S. epidermidis* MFP04 and confirmed the absence of significant variations in the expression of potential cytotoxic proteins (Table S1).

### CGRP affects the surface properties of *S. epidermidis*

**Figure 7:**
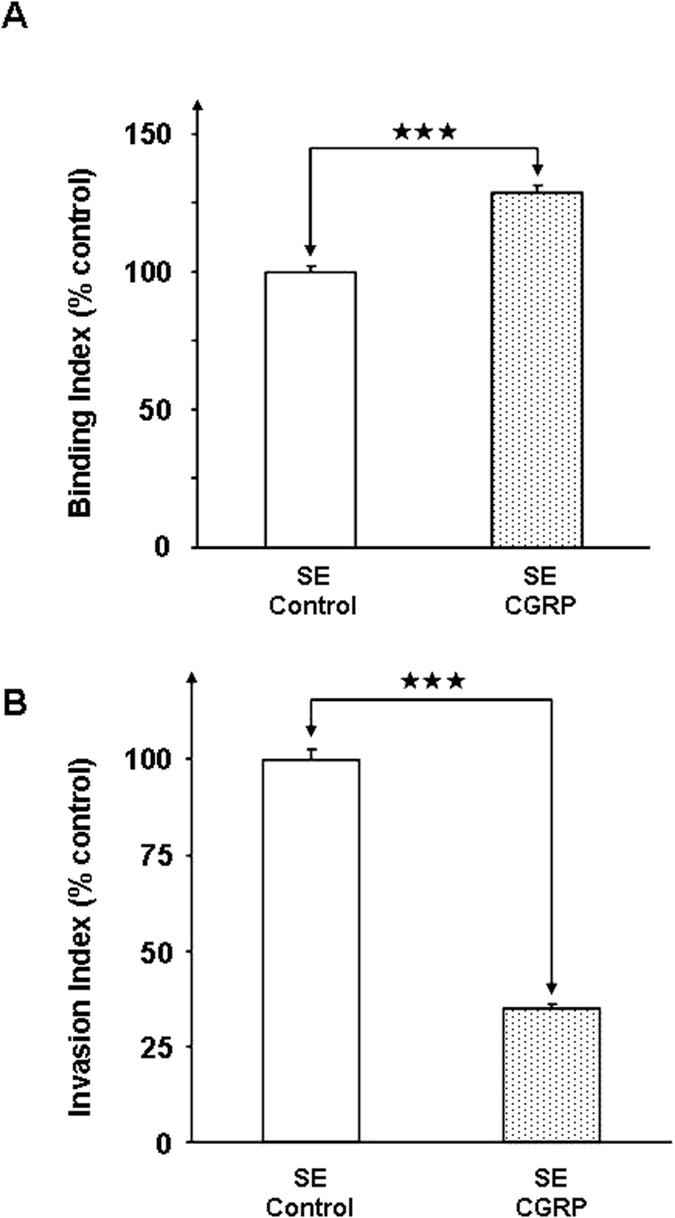
Effects of CGRP (10^−6 ^M) on the binding (**A**) and invasion activities (**B**) of *S. epidermidis* (SE) toward HaCaT cells. After 1 h incubation at a MOI of 10:1, the total number of cell-associated bacteria was determined by direct counting after plating. Invasive bacteria were also quantified by direct counting after elimination of extracellular bacteria by gentamicin. (^★★★^*p* < 0.001). The results are representative of three independent experiments.

**Figure 8:**
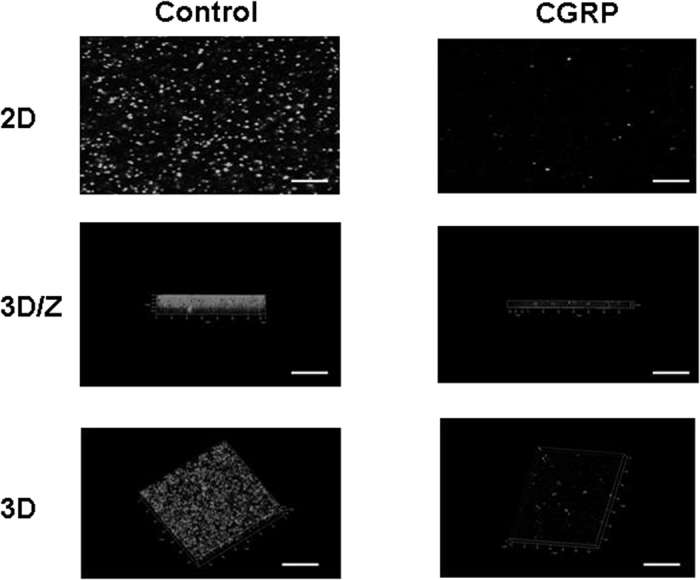
Effect of CGRP (10^−6 ^M) on *S. epidermidis* biofilm formation under dynamic conditions. Biofilm formation by *S. epidermidis* in the absence or presence of CGRP was studied by confocal laser scanning microscopy using a flow-cell system. Two dimensional (2D) images collected at 1 μm intervals were used to reconstruct ortho cuts (3D/z) and three-dimensional (3D) images. Pictures are representative of three independent experiments. Scale bars = 20 μm.

**Figure 9:**
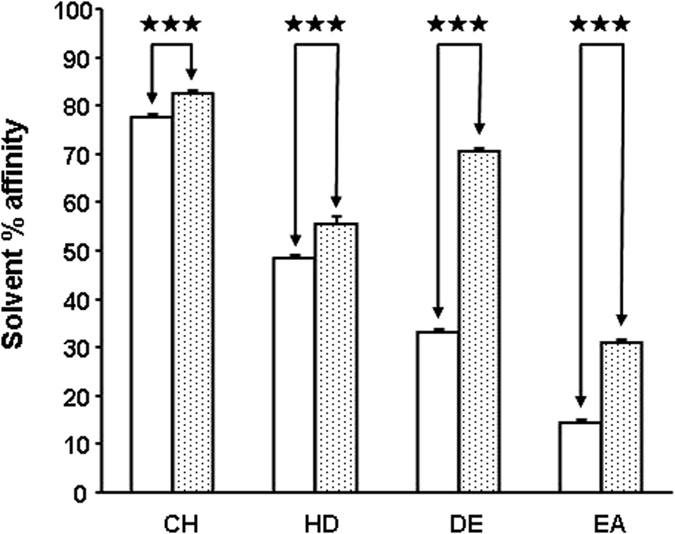
Effects of CGRP (10−6 M) on the relative affinity of S. epidermidis to solvents. Partition between water and solvents of different polarities and Lewis acid-base values of control (empty bars) and CGRP-treated (dotted bars) *S. epidermidis* was studied with the MATS technique using chloroform (CH), hexadecane (HD), decane (DE) and ethyl acetate (EA). (^★★★^*p* < 0.001). The results are representative of three independent experiments.

Adhesion and internalization of *S epidermidis* were studied using HaCaT cells and a gentamicin protection assay, as previously described^18^. When bacteria were pre-treated with CGRP (10^−6^ M) a significant increase in *S. epidermidis* adhesion to the surfaces of keratinocytes was observed (+29 ± 3%, *p* < 0.001) (Fig. 7A). This effect was associated with a marked decrease in *S. epidermidis* internalization in HaCaT cells (−65 ± 4%, *p* < 0.001) (Fig. 7B). The effect of CGRP on biofilm formation by *S. epidermidis* was investigated by confocal laser-scanning microscopy in a flow cell system. When bacteria were pretreated with CGRP and introduced into the flow cell system, a marked decrease in biofilm formation was observed (Fig. 8). After 24 h, the mean thickness of the biofilm of control bacteria reached 23 ± 1 μm, whereas it was only of 6 ± 1 μm with CGRP-treated *S. epidermidis*. The calculated biomass showed a similar significant decrease (−68 ± 16, *p* < 0.01%). These effects were correlated with a clear evolution of the surface polarity of CGRP-treated bacteria, as measured by the MATS technique (Microbial Adhesion To Solvents^19^). The higher affinity for apolar solvents (decane and hexadecane - Fig. 9) indicated that after exposure to CGRP, the surface of the bacterium evolved from having a mid-hydrophilic to a low-hydrophobic characteristic. In parallel, the affinity of the bacterium for hexadecane and chloroform, related to the Lewis acid characteristic, was marginally increased (+6 ± 1 and +15 ± 4%, *p* < 0.001, respectively) whereas the affinity for decane and ethyl acetate, showing Lewis acid characteristics, was markedly enhanced (+113 ± 4 and +115 ± 7%, *p* < 0.001, respectively). Thus the Lewis acid characteristic of the bacterial surface was reinforced.

### The DnaK protein acts as a sensor of CGRP in *S. epidermidis*

**Figure 10:**
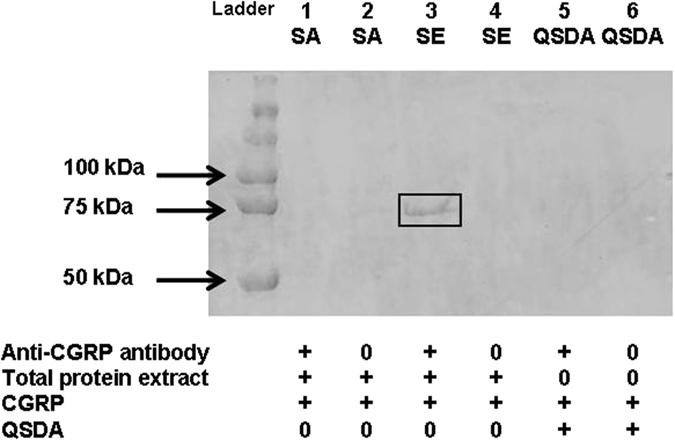
SDS page analysis of CGRP-binding proteins in *S. aureus* and *S. epidermidis* membrane extracts. The CGRP sensor was identified in *S. aureus* (SA) and *S. epidermidis* (SE) membrane extracts by immunoprecipitation using CGRP antibody-associated beads. CGRP was associated with a 70 kDa apparent mass protein that was identified by tandem mass spectrometry as the chaperone protein DnaK. The lactonase QSDA was used as a control. The results are representative of three independent experiments.

Assuming that CGRP is unable to cross the bacterial membrane because of its charge and hook shape^20^ and the binding of CGRP to its sensor protein is sufficiently stable, we investigated the CGRP-binding protein in *S. epidermidis* membrane extracts via an immunoprecipitation technique using CGRP antibody-coated beads as previously described^5^. A unique CGRP binding protein of an apparent mass of 70 kDa was identified in the proteome of *S. epidermidis* MFP04 (Fig. 10). In agreement with the absence of an effect of CGRP on *S. aureus*, this protein was absent in the membrane proteome in *S. aureus* MFP03 (Fig. 10). A band corresponding to this protein was subsequently dissected and analyzed by tandem mass spectrometry. This protein was identified by mass spectrometry analysis with a score of 140.29 and coverage of 64 as the 66.1 kDa chaperone protein of *S. epidermidis* DnaK.

### Export of DnaK through the large conductance mechanosensitive channel MscL is required for the effects of CGRP on *S. epidermidis*

**Figure 11:**
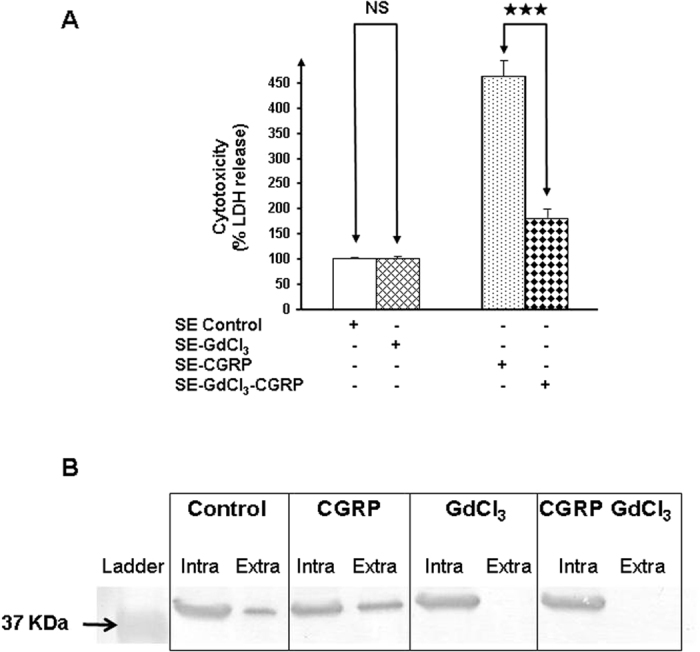
Effect of pre-treatment by GdCl_3_ on the cytotoxicity of control and CGRP- treated *S. epidermidis* (**A**) and on the relative intra- and extra-cellular concentrations of EfTu produced by *S. epidermidis* in control conditions and after exposure to CGRP, GdCl_3_ or CGRP and GdCl_3_ (**B**). Bacteria (SE) exposed or not to GdCl_3_ (1 mM) were subsequently grown in the absence or presence CGRP (10^−6 ^M). Their cytotoxic potential was measured on HaCaT cells (**A**). EfTu was analyzed by western blotting in the stroma (intra) and growth medium (extra) of *S. epidermidis* grown in the absence (control) or presence of CGRP (10^−6 ^M), GdCl_3_ (1 mM) or both substances (CGRP and GdCl_3_) (**B**). (NS: non significant; ^★★★^*p* < 0.001). The results are representative of three independent experiments.

Electrophysiological approaches have demonstrated that in the membrane of *E. coli*, DnaK is exported through the large conductance mechanosensitive channel MscL^21^. MscL also triggers the export of EfTu^21^, the Substance P sensor protein in *S. epidermidis*^6^. MscL is a ubiquitous protein and is also expressed by *S. epidermidis*^22^. As shown herein, CGRP increases the export of DnaK by *S. epidermidis* (Spot 7 in Fig. 6 and Table 1). The effect of CGRP on *S. epidermidis* cytotoxicity was then studied in the presence of GdCl_3_ (10^−3 ^M) a specific inhibitor of the MscL channel^23^. Exposure of bacteria to GdCl_3_ resulted in a significant reduction of the cytotoxic activity of CGRP-treated bacteria (−78 ± 15%, *p* < 0.001) (Fig. 11A). In contrast, the effect of the control bacteria remained unchanged, suggesting that in the absence of CGRP, DnaK is unable to modulate bacterial cytotoxicity. In parallel, we observed through western blotting that CGRP induces a concomitant export of EfTu (Fig. 11B). Moreover, EfTu translocation through the bacterial membrane was completely inhibited by exposure to GdCl_3_ (10^−3 ^M) (Fig. 11B). This inhibition was observed in either the absence or presence of CGRP.

## Discussion

Skin is a complex neuroendocrine organ whose physiology results from equilibrium among many local factors. In addition to the well-documented release of Substance P, skin sensory nerve terminals generally release CGRP^1^. This neuropeptide is also expressed locally by monocytes and macrophages^24^, Langerhans cells^25^ and keratinocytes^26^. CGRP is produced in significant amounts in skin and is now recognized as a pleiotropic signaling molecule in mammals^27^. The present work extends this notion, showing that CGRP can also act as a regulator of bacterial physiology, modulating the virulence and surface properties of one of the principle skin associated bacteria, *S. epidermidis*.

The stimulatory effect of CGRP on *S. epidermidis* cytotoxicity is stronger than that observed under identical doses of Substance P^5^. Similarly, the threshold of CGRP activity on *S. epidermidis* is extremely low, below the picomolar level, and well below the mean concentrations of CGRP in blood (±84 × 10^−12 ^M)^28^ and skin (between 5.4 and 0.3 × 10^−12 ^M/g tissue)^10^. The effect of CGRP on *S. epidermidis* virulence was confirmed using RHE. RHE shows a fully differentiated stratum corneum^29^, thus indicating that this observation is physiologically relevant. The low concentration of CGRP required to induce a bacterial response allows metabolic action of the peptide to be excluded. Moreover, the absence of the effect of CGRP on *S. aureus* indicates that it is highly specific, because this species is closely related to *S. epidermidis.* In contrast, the antagonistic effect of CGRP and Substance P towards *S. epidermidis* suggests the existence of a common step in the response of the bacterium to both peptides. This hypothesis was confirmed later in this study.

In agreement with these first results, we observed that CGRP-treated bacteria induce an up-regulation of interleukin 8 (IL8) secretion by cultured keratinocytes and RHE. In skin, as in many tissues, this result indicates the activation of an inflammatory process^30^. Because the pro-inflammatory response of keratinocytes is generally associated with induction of antimicrobial peptide secretion^16^, the production of LL37 and HBD2 by HaCaT cells and RHE was measured after exposure to CGRP-treated bacteria. An increase in LL37 secretion was observed in both models when these cells were exposed to CGRP pre-treated *S. epidermidis. S. epidermidis* is one of the major commensal bacteria of the human skin and is normally well tolerated^31^. However, the present results demonstrate that when the bacterium is exposed to CGRP, the skin tolerance towards it is reduced, and an innate immune response is activated. Nevertheless, this response should be incomplete because a parallel decrease in HBD2 production was observed.

Analysis of the secretome of CGRP-treated bacteria by two techniques, 2D electrophoresis coupled with MALDI-TOF/TOF and tandem mass spectrometry, did not reveal significant variations in the production of diffusible proteins/enzymes with similarity to known virulence factors. The IsaB protein and its precursor were increased, but IsaB is involved in the escape mechanism from the host defense system^32^ and is not directly responsible for bacterial cytotoxicity. Of course, we cannot exclude the association of several individual proteins among the 170 regularly identified in the secretome of *S. epidermidis* MFP04 (this study); however, even in this list, the number of proteins or enzymes potentially capable of acting as virulence factors is very limited. In contrast, an increase in several chaperone proteins involved in bacterial adaptation was observed, suggesting that CGRP is detected by *S. epidermidis* as a stress signal. In fact, CGRP appears to modulate the surface properties of *S. epidermidis*. The bacterium showed increased adherence to keratinocytes. In contrast, its internalization process was reduced, thus suggesting that the surface properties of the bacterium hindered its penetration into the cytoplasmic compartment of the target cell. *S. epidermidis* internalization is mediated by the major autolysin/adhesin AtlE, and the eukaryotic protein Hsp70 was identified as its putative host cell receptor^33^. However, in the present study we observed that CGRP induced an increase in the export of DnaK, a member of the heat shock protein 70 (Hsp70) family^34^. Thus, we can not exclude the possibility that DnaK might interfere with AtlE on the *S. epidermidis* surface, inducing an inhibition of its adhesion potential toward target cells. In *Staphylococci*, DnaK is also involved in biofilm formation^35^ and thus is a potential target for new antibiofilm molecules^36^. In agreement with the hypothesis of a key role of DnaK in the response of *S. epidermidis* to CGRP, we observed that the biofilm formation activity of the bacterium was reduced after exposure to the peptide. In the present study, the biofilm formation was monitored on a glass surface and under dynamic conditions. Glass is a polar surface^37^, and bacteria must develop sufficient adhesion forces to resist the flow. Thus, our observations suggest that CGRP-treated bacteria present a decrease in adherence to polar materials such as glass and thus present a more hydrophobic surface. This hypothesis was confirmed through use of the MATS technique, which revealed that after exposure to CGRP, the surfaces of the bacteria evolved from having a mid-hydrophilic to a low-hydrophobic characteristic. These results are also coherent with the higher adhesion on the surface of HaCaT cells, thus suggesting that CGRP-treated bacteria develop a higher affinity toward membrane phospholipids than do untreated bacteria. Although no direct relationship was found among surface polarity, biofilm formation and virulence in clinical species of *S. epidermidis*^38^, CGRP appears to have has a coherent effect on these parameters, thus leading to increased virulence.

**Figure 12:**
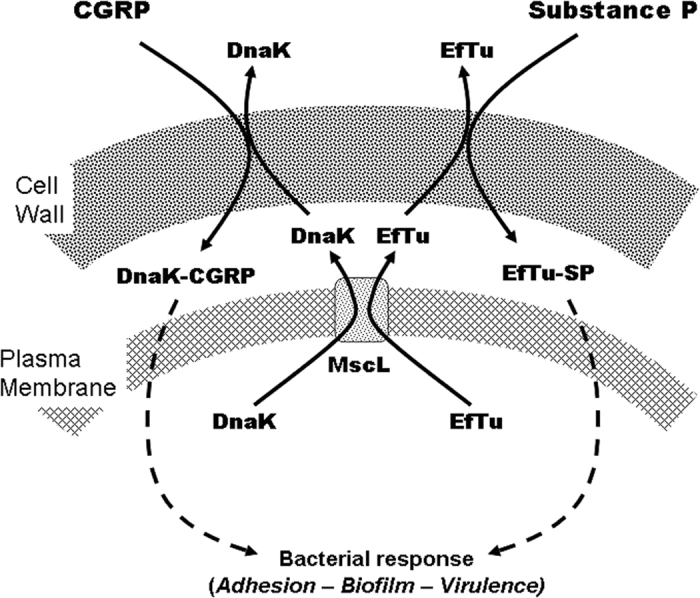
Schematic model representing the potential translocation of DnaK and EfTu through the membrane of *S. epidermidis*, thus leading to recognition of CGRP and Substance P. DnaK and CGRP are probably translocated in the periplasmic compartment through the MscL channel. CGRP and Substance P are binding to DnaK and EfTu, either in the periplasm or after diffusion through the cell wall, thus leading to formation of complexes able to promote the bacterial response through a yet-unknown mechanism. Competition of DnaK and EfTu for the same transporter may explain the antagonistic effects of CGRP and Substance P.

A central role of DnaK in the response of *S. epidermidis* to CGRP was confirmed when this protein was identified by immunoprecipitation and tandem mass spectrometry as the CGRP binding protein. In *Staphylococci*, DnaK is known for its multiple roles in environment and stress adaption^35^ but the binding of CGRP to DnaK was initially unexpected. However, alignment of the *S. epidermidis* DnaK amino acid sequence with eukaryotic sequences shows matches at the first rank with protein Q8T885 [www.uniprot.org] which is also designated as a CGRP receptor component protein (CGRP-RCP), a subunit of the CGRP receptor of eukaryotes necessary for signal transduction^39^. Moreover, studies in yeast suggest that CGRP-RCP is a multifunctional protein involved in transcription^40^, and in a recent study in *S. epidermidis* DnaK has been suggested to be exposed at the surface of the bacterium and to act as a receptor for endothelial cells^33^. A STRING10 network analysis [http://string-db.org/] indicated that *S. epidermidis* DnaK interacts not only with other chaperone proteins but also with EfTu, the moonlighting protein previously identified as the sensor for Substance P in *Staphylococci*^6^ (Figure S1). In *E. coli*, DnaK and EfTu are released upon osmotic shock by a translocation mechanism involving the mechanosensitive channel MscL^21^. MscL can be blocked by gadolinium chloride (GdCl_3_)^23^, and we observed that pre-exposure of *S. epidermidis* to GdCl_3_ inhibited the stimulatory effect of CGRP on *S. epidermidis* cytotoxicity. In addition, CGRP increases the release of EfTu, and this effect is antagonized by GdCl_3_, thus suggesting that the peptide induces the opening of MscL and, as shown in *E. coli,* a simultaneous translocation of DnaK and EfTu^21^. Of course the increase of DnaK in the secretome of CGRP-treated bacteria could potentially result from many other events, including increased membrane permeability, cell lysis or reduced extracellular degradation. However, in MscL-deficient strains of *E. coli,* DnaK is not released, even after osmotic shock^21^, suggesting that DnaK export is almost exclusively controlled by MscL. Because we observed that CGRP did not affect the growth and viability of *S. epidermidis* and that as a chaperone, DnaK is a stable molecule, the effect of CGRP on DnaK export through MscL channels is the main hypothesis explaining the present results. Moreover, this hypothesis provides a potential explanation for the antagonistic effects of CGRP and Substance P. Indeed, if DnaK and EfTu are exported through the same channel, they would compete for translocation when the bacterium is simultaneously exposed to CGRP and Susbtance P. This hypothesis is summarized in Fig. 12. This mechanism may also explain the absence of sensitivity of *S. aureus* to CGRP. Indeed, DnaK is also expressed in S*. aureus*, but the oligomeric structure of MscL is still a matter of debate, and the diameter of this channel differs among bacterial species^41^. EfTu is a 43 kDa protein but DnaK is much larger (66.1 kDa) and it is possible that DnaK is unable to translocate through the *S. aureus* membrane, thus making this species insensitive to CGRP.

Reasons for the preservation or emergence of a CGRP-sensing system in *S. epidermidis* remain hypothetical. However, interestingly *S. epidermidis* not only is a commensal bacterium but also is associated with severe forms of atopic dermatitis^42^ and is currently regarded as the most frequent cause of nosocomial and medical device-associated infections^43^. CGRP expression is increased during stress and in psoriatic skin and this peptide is one of the most potent vasodilatory mediators^1^. Because, in parallel CGRP reduces neutrophilic accumulation^1^, as has been described in the lung, where *Pseudomonas aeruginosa* uses interferon as an infection inducer^44^, *S. epidermidis* should detect CGRP as a signal of favorable conditions for tissue invasion and shift from commensal to pathogenic behavior.

In conclusion, this study reveals a totally new function of CGRP in the skin as a regulator of *S. epidermidis* virulence. Our results also illustrate a new role of DnaK as a moonlighting protein^45^ acting as a sensor for CGRP in *S. epidermidis*. The translocation mechanism of DnaK through MscL provides an explanation for the antagonistic effects of CGRP and Substance P on *S. epidermidis* and for the absence of the effect of CGRP on *S. aureus*. These results demonstrate that skin bacteria can integrate different signals from cutaneous origin and consequently adapt their virulence to the microenvironment.

## Methods

### Bacterial strains and culture conditions

*S. aureus* (MFP03) and *S. epidermidis* (MFP04) were collected from the skin of healthy volunteers^46^. These bacteria were characterized by phenotypic, metabolic, MALDI-Biotyper proteomic analysis and 16S ribosomal RNA gene sequencing. All bacteria were grown at 37 °C in Luria-Bertani (LB) medium under gentle agitation (180 rpm). Bacteria were stored on cryobeads at −140 °C and subjected to two pre-culture phases. For the studies, bacteria collected at the end of the exponential growth phase were diluted in fresh broth. The peptides, diluted in sterile physiological water (NaCl 0.9%), or an equivalent volume of physiological water in control studies, were added at the beginning of the log growth phase. Before the tests, the bacteria were harvested by centrifugation (7,000 × *g*) and washed with sterile physiological water to remove any trace of free peptide. The bacterial density and the absence of contamination were controlled by plating. The viability of the bacteria in eukaryotic cell culture medium and under different culture conditions was controlled in preliminary studies (*data not shown*). CGRP and Substance P were obtained from Polypeptides (Strasbourg, France). Gadolinium chloride (GdCl_3_) was obtained from Sigma-Aldrich (Saint-Quentin Fallavier, France).

### Cytotoxicity studies

The cytotoxicity of bacteria was studied in the human keratinocyte cell line HaCaT and in RHE. HaCaT Cells, provided by Cell Line Services (Eppelhein, Germany), were grown at 37 °C under a 5% CO_2_ atmosphere, in Dulbecco’s modified Eagle’s medium (DMEM, Lonza, Levallois-Perret, France) supplemented with 10% fetal calf serum and 1% antibiotic cocktail (HyClone Thermo Scientific, Illkirch, France). Cells were used between passages 41 and 65. One day before use, the HaCaT cells were starved of antibiotic and fetal calf serum. The cells were incubated with control or treated bacteria at a multiplicity of infection (MOI) of 10:1.

RHE, obtained from Episkin (Lyon, France) was grown according to the specifications of the provider. Briefly, after delivery RHE was immediately incubated at 37 °C, under a 5% CO_2_ atmosphere, in provider medium and allowed to stabilize for 48h. One day before use, RHE was transferred to antibiotics-free medium. For infection tests, RHE was exposed to control or treated bacteria at a final concentration of 10^6^ or 10^7^ CFU.

The lethal effect of bacteria was determined by assaying LDH which is released by keratinocytes, by using a Cytotox 96 assay (Promega, Charbonnières, France) and by measurement of keratinocytes survival with MTT assays, thus allowing cellular NAD(P)H-dependent metabolic activity to be determined through evaluation of the conversion of a tetrazolium dye (MTT: 3-(4,5-dimethylthiazol-2-yl)-2,5-diphenyltetrazolium bromide) into insoluble purple formazan crystals according to the OECD Draft Revised Guideline TG431^15^.

### Antimicrobial peptides and interleukin 8 assays

The potential effect of CGRP on *S. epidermidis*-induced expression of antimicrobial peptides and chemokines was investigated by measurement of IL8, cathelicidin (LL37) and β-defensin 2 (HBD2) production by HaCaT cells and RHE after exposure to control or CGRP-treated bacteria. IL8, LL37 and HBD2 were assayed in the culture medium of HaCaT cells and RHE by using human CXCL8/IL-8 cat. D8000C (R&D system, Lille, France), human cathelicidin cat. CSB-EL004476HU and β-defensin 2 cat. CSB-E13201h (Cusabio, Aachen, Germany) ELISA kits according to the manufacturers’ protocols.

### Secretome analysis

Supernatants of control or CGRP-treated *S. epidermidis* were obtained by centrifugation and filtration. Proteins were precipitated by the addition of trichloroacetic acid on ice. These proteins were harvested by centrifugation, washed in cold acetone, dried at room temperature and re-dissolved in a rehydratation buffer^47^. The protein concentration was determined by Bradford assays. Equal amounts of proteins (300 μg) were loaded on each gel (12% w/v polyacrylamide) and separated. For 2D-gel electrophoresis, protein samples were first separated by isoelectric focusing (IEF) using pH 3 to 10 nonlinear IEF strips (GE Healthcare, Vélizy-Villacoublay, France) as previously described^47^. The strips were then transferred horizontally onto 12% polyacrylamide gels and covered with 0.5% agarose and the second dimension separation was run. Proteins were visualized by colloidal Coomassie brilliant blue G250 staining. Gel images were captured using a GS-800 densitometer (Bio-Rad, Schiltigheim, France). Variations in spot intensity and distribution were studied using Bio-Rad PDQuest 2D^®^ analysis software. Culture medium proteins were excluded and electrophoretic spots of interest were dissected and subjected to in-gel trypsin digestion and analyzed by MALDI-TOF/TOF using an AutoFlex III mass spectrometer (Bruker Daltonics, Wissembourg, France). The spectrometer was used in a positive/reflector mode. Samples were spotted to MTP 384 ground steel targets (Bruker Daltonics, Wissembourg, France) using a freshly prepared matrix solution composed of 2,5-dihydroxybenzoic acid in a solution of trifluoroacetic acid and acetonitrile. Each spectrum was established over an average of 500–1000 laser shots. FlexAnalysis software generated an MS peak list, which was subjected to peptide mass fingerprinting using the integrated software Biotools (Version 3.2). The NCBI database was searched using the online MASCOT software and statistical sequence analyses were performed using the probability-based Mowse score.

For tandem mass spectrometry analysis, all experiments were performed using a LTQ-Orbitrap Elite coupled with an Easy nLC II system (Thermo Scientific, Villebon-sur-Yvette, France). The samples were injected onto an enrichment column (C18 PepMap100, Thermo Scientific, Villebon-sur-Yvette, France). Separation was achieved with an analytical column needle (NTCC-360/100-5-153, NikkyoTechnos, Tokyo, Japan). The mobile phase consisted of H_2_O/0.1% FA (buffer A) and ACN/FA 0.1% (buffer B). Tryptic peptides were eluted at a flowrate of 300 nL/min using a three-step linear gradient: from 2 to 40% B over 25 min. The mass spectrometer was operated in positive ion mode with a capillary voltage and a source temperature set at 1.5 kV and 275 °C, respectively. The samples were analyzed using the CID (collision induced dissociation) method. The first scan (MS spectra) was recorded in the Orbitrap analyzer (R = 60,000) in the mass range m/z 400–1800. The 20 most intense ions were then selected for MS2 experiments. Singly charged species were excluded for MS2 analysis. Dynamic exclusion of already fragmented precursor ions was applied for 30 s, with a repeat count of 1, a repeat duration of 30 s and an exclusion mass width of ±10 ppm. Fragmentation occurred in the linear ion trap analyzer with collision energy of 35. All measurements in the Orbitrap analyzer were performed with on-the-fly internal recalibration (lock mass) at m/z 445.12002 (polydimethylcyclosiloxane). Raw data files were processed using Proteome Discoverer 1.3 software (Thermo Scientific, Villebon-sur-Yvette, France). Peak lists were searched using the MASCOT search engine (Matrix Science, Boston, USA) against the database Swiss Prot database searches were performed with the following parameters: 1 missed trypsin cleavage site allowed; variable modifications: carbamidomethylation of cystein, and oxidation of methionine; mass tolerance on parent and daughter ions: 10 ppm and 0.5 Da respectively. Only proteins represented by more than 1 peptide sequence, by 2 folds and with power >0.8 were retained. Each protein identification was validated by an ANOVA test.

### Binding and invasion studies

Binding and invasion studies were performed with HaCaT cells. Cultured keratinocytes were infected for 1 h with *S. epidermidis* (MFP04) at an MOI of 10:1. In the first series of experiments, at the end of the incubation period HaCaT cells were washed six times with DMEM to remove non-adherent bacteria and then disrupted with 1 mL of 0.9% Triton X100. The total number of cell-associated bacteria (intra- and extra-cellular) was then counted by plating serial dilutions on Tryptone Soya Agar medium (TSA). Invasive bacteria were quantified by gentamicin protection assays as described by Mezghani-Abdelmoula *et al.*^18^. In these experiments, at the end of the incubation period HaCaT cells were treated with 100 μg/mL gentamicin to kill extracellular bacteria. The cells were then washed and lysed as previously described and the number of invading bacteria released from the cells was counted. Cells surface adherent bacteria were calculated by subtracting of the number of invasive bacteria from the total number of cell-associated bacteria. For each assay, serial dilutions of the whole bacterial inoculum were plated. The effect of HaCaT cell lysis buffer on the viability of *S. epidermidis* (MFP04) was controlled in preliminary tests.

### Biofilm formation studies under dynamic conditions

After an overnight pre-culture in LB at 37 °C, *S. epidermidis* MFP04 was inoculated at an OD_580_ of 0.08 in LB medium and sub-cultured for 2 h. CGRP (10^−6 ^M final concentration) was added, and bacteria were grown for an additional 1 h. Bacteria were then washed and adjusted to an OD_580_ of 0.1 in 0.9% NaCl supplemented with CGRP (10^−6 ^M). The bacterial suspensions were then used to study biofilm formation under dynamic conditions at 37 °C in a three-channel flow cell as described by Bazire *et al.*^48^. Briefly, each bacterial suspension was injected into a flow cell channel, and bacteria were allowed to adhere to the glass coverslip for 2 h. A flow (2.5 mL/h) of LB medium was then applied for 24 h. At the end of the experiments, the biofilms were stained with 5 × 10^−6 ^M Syto 61 red dye or 5 × 10^−6 ^M SytoX (Molecular Probes, Thermo Fisher Scientific, Villebon-sur-Yvette, France). Observations were made using a confocal laser scanning microscope (LSM 710 confocal laser-scanning microscope; Zeiss, Marly le Roi, France). The biofilm thicknesses and corresponding biovolumes were estimated by measuring field samples from at least 3 independent experiments using COMSTAT software (Matworks, Natick, USA)^49^.

### Characterization of bacterial surface polarity

Stationary phase bacteria were harvested (7,000 × *g*; 10 minutes) and washed twice in 0.9% NaCl. The polarity of control and CGRP-treated *S. epidermidis* MFP04 was studied using the Microbial Adhesion To Solvent (MATS) technique^19^ and two solvent couples: chloroform/hexadecane and ethyl acetate/n-decane. In total, 2.6 mL of bacterial suspension at OD_400_ 0.8 was mixed for 60 s with 0.4 mL of each solvent indicated above. The OD of the aqueous phase was measured at 400 nm. The percentage of cells in each solvent was calculated using the following equation: (1−A/A_0_) × 100. Each experiment was repeated in triplicate using three independent cultures.

### CGRP binding site identification

Mid-log growth phase bacterial cultures were centrifuged at 7,000 × *g* for 10 min. The pellets were resuspended in 5 mL of lysostaphin diluted in phosphate buffered saline (PBS) (10^−5 ^g/mL). This mix was incubated for 1h at 37 °C with shaking at 180 rpm. Then, the samples were then centrifuged for 20 min at 7,000 × *g* and 4 °C. Each pellet was solubilized in 6 mL of non-denaturing lysis buffer (Tris 50 × 10^−3 ^M pH 8, EDTA 4 × 10^−3 ^M, NaCl 137 × 10^−3 ^M, glycerol 10%, Triton X100 1%, phenylmethylsulfonyl-fluoride 10^−3 ^M and supplemented with protease inhibitors (Boehringer, Paris, France). Four freeze/thaw cycles were applied to the suspension at −80 °C for 20 min/37 °C for 10 min. The bacteria were lysed by sonication using a number of short pulses (1 min) with pauses (2 min) on ice to maintain a low temperature. The cell lysates were once again centrifuged at 13,000 × *g* for 10 min at 4 °C to remove unbroken cells. For total protein extracts, the supernatant was incubated with benzonase (0.125 × 10^−6 ^L/mL) and MgCl_2_ (1.625 × 10^−6 ^L/mL) for 1 h at room temperature to degrade nucleic acids. The proteins were precipitated with 20% trichloroacetic acid, vortexed for 15 seconds and incubated overnight at 4 °C. Samples were then centrifuged at 13,000 × *g* for 20 min at 4 °C and solubilized in a rehydratation buffer (urea 7 M, 15.2% thio-urea, 4% CHAPS, 25 × 10^−6 ^L/mL IPG buffer, 10.79 × 10^−3 ^g/mL DTT and 1.43 10^−3 ^g/mL TCEP in water). To extract membrane proteins, the supernatant containing the total proteins was ultracentrifuged at 35,000 rpm for 50 min at 4 °C. The pellet containing membrane proteins was solubilized in a solution containing 5 × 10^−5 ^M Tris pH 8, 10^−5 ^M MgCl_2_, 2% Triton X100 and 10^−9 ^M phenylmethylsulfonylfluoride and supplemented with protease inhibitors.

The CGRP binding site was extracted by using an immunoprecipitation technique adapted from Mijouin *et al.*^5^. Briefly, 6 × 10^−6 ^L of G protein-coupled agarose beads (Millipore) and 1.5 10^−6 ^g of CGRP monoclonal antibodies (Abcam, Paris, France) were allowed to associate overnight at 4 °C. Meanwhile, 1.5 × 10^−3 ^g of membrane protein extracts was incubated with CGRP (10^−6 ^M) for 1h at room temperature under slow orbital agitation. To reduce the non-specific binding, 5 × 10^−5 ^L of rabbit polyclonal serum was added. Unlabelled G protein-coupled agarose beads were added to remove non-specific complexes. The samples were incubated for 30 min at 4 °C under slow orbital agitation. The unlabelled beads were removed by centrifugation (10,000 × g, 4 °C, 10 min). The supernatant containing the CGRP bound ligand was then mixed with the CGRP antibody-associated beads and incubated for 1 h at room temperature under slow orbital agitation. For each sample, the beads were washed two times with 250 × 10^−6 ^L of non-denaturing lysis buffer by low-speed centrifugation cycles. Each sample was boiled for 5 min with 5 × 10^−5 ^L of Laemmli 2x buffer (Tris 0.2 M pH 6.8, glycerol 45%, sodium dodecyl sulfate (SDS) 6%, β-mercaptoethanol 6% and bromophenol blue 0.03%) to separate the ligand from the beads. Before loading on a 12% SDS polyacrylamide gel electrophoresis (PAGE) gel, the beads were removed by centrifugation (14,000 × *g*, 4 °C, 5 min). Proteins were visualized by colloidal Coomassie blue G250 staining (Sigma-Aldrich, Saint Quentin Fallavier, France). Three independent experiments were conducted.

After dissection of the band of interest and protein solubilization, the CGRP-binding proteins were identified by tandem mass spectrometry as previously described, using an LTQ-Orbitrap Elite coupled with an Easy nLC II system (Thermo Scientific, Villebon-sur-Yvette, France).

### Western blot analysis of EfTu in intra- and extra-bacterial compartments

Proteins extracted from the bacterial stroma and growth medium after fractionation as previously described were separated on a 12% w/v polyacrylamide SDS-PAGE gel. After separation, proteins were transferred onto nitrocellulose membranes at 50 mA for 1h in Tris base (0.192 M) transfer buffer containing glycine (14.5% g/L w/v) and methanol (20% v/v) using a Bio-Rad Mini TransBlot Electrophoretic Transfer Cell system. Membranes were then air-dried and immersed for 2 h in blocking buffer (Tris buffer saline (TBS): 5 × 10^−5 ^M, 0.15 M NaCl, 5% whole milk). Membranes were incubated with primary antibody raised against bacterial EfTu^6^ (1:100 in blocking buffer) at room temperature for 2 h while shaking. After incubation, the blot was washed three times in 1x TBS supplemented with 0.05% (w/v) Tween 20 for 30 min and incubated for 1.5 h with shaking with secondary antibody diluted 1/5000 (goat anti-rabbit IgG alkaline phosphatase conjugate, Bio-Rad, Schiltigheim, France).). Electrophoretic bands were detected using an alkaline phosphatase conjugate substrate kit (Bio-Rad, Schiltigheim, France).

### Statistical analysis

All results are expressed as means ± standard error (SEM) calculated over a minimum of three independent experiments. Significant differences were estimated using Student’s *t* tests and are noted as ★, ★★ and ★★★ for *p*-values < *0.05*, < *0.01* and < *0.001*, respectively. For confocal microscopy studies, the biofilm thickness and biomass were calculated from a minimum of 20 measures in different fields.

## Additional Information

**How to cite this article**: N’Diaye, A. R. *et al.* Skin-bacteria communication: Involvement of the neurohormone Calcitonin Gene Related Peptide (CGRP) in the regulation of *Staphylococcus epidermidis* virulence. *Sci. Rep.* **6**, 35379; doi: 10.1038/srep35379 (2016).

## Supplementary Material

Supplementary Information

## Figures and Tables

**Table 1:** Proteins over-expressed in the secretome of CGRP-treated *S. epidermidis* identified by MALDI-TOF/TOF.

Spot	NCBI accession Number	Gene Name	Putative function	Mascot score	Number of matched peptides	Coverage (%)	Mass (Da) & pI
1	WP_002435425.1	Dihydrolipoyl dehydrogenase	Pyridine nucleotide-disulphide oxidoreductase fold NAD(P)(+)-binding proteins	107	16	41	49733/4.72
2	WP_002434485.1	Inosine-monophosphate dehydrogenase	The catalytic domain of the inosine-monophosphate dehydrogenase (IMPDH). IMPDH catalyzes the NAD-dependent oxidation of inosine 5′-monophosphate (IMP) to xanthosine 5′monophosphate (XMP)	100	21	43	52493/5.35
3	WP_002453341.1	Superoxidase dismutase	Inorganic ion transport and metabolism	108	15	71	22697/5.04
4	WP_002435927.1	Immunodominant surface antigen B (isaB)	Immune response during septicemia: generally classified as a virulence factor (increased expression *in vivo* during human sepsis)	98	10	62	20088/4.84
5	WP_002432778.1	Alkaline shock protein 23	Alkaline pH tolerance	120	18	72	19187/4.91
6	WP_002434679.1	Citrate synthase	CoA binding site: oxalate citrate binding site catalytic triad	144	24	55	42519/5.26
7	WP_049427550.1	DnaK	Chaperone: functions in stress- induced protein refolding and degradation	242	43	72	66071/4.54
8	WP_002436880.1	Immunodominant antigen B (isaB) precursor	IsaB pro-protein	101	8	41	18400/5.54
9	WP_030061284.1	Peroxidoxin	Controls cytokine-induced peroxide levels: confers protection to cells	141	13	91	21268/4.5
