# Supplementary material for: Skin-bacteria communication: Involvement of the neurohormone Calcitonin Gene Related Peptide (CGRP) in the regulation of Staphylococcus epidermidis virulence
Source: Sci Rep. 2016 Oct 14;6:35379. doi: 10.1038/srep35379 (PMC5064375; doi:10.1038/srep35379)
Supplement: Supplementary Information [file srep35379-s1.doc]

**Skin-bacteria communication: Involvement of the neurohormone Calcitonin Gene Related Peptide (CGRP) in the regulation of *Staphylococcus epidermidis* virulence**

**Awa R. N’Diaye^1^, Camille Leclerc^1^, Takfarinas Kentache^2^, Julie Hardouin^2^, Cecile Duclairoir Poc^1^,** **Yoan Konto-Ghiorghi^1^, Sylvie Chevalier^1^, Olivier Lesouhaitier^1^, Marc G.J. Feuilloley^1*^**

**1** Laboratory of Microbiology Signals and Microenvironnement, LMSM, EA 4312, Normandie Université, Evreux, France

**2** Laboratory of Polymers, Biopolymers and Surfaces, CNRS UMR 6270, Normandie Université, Mont-Saint-Aignan, France

**Figure S1. Interaction network of DnaK generated by STRING10 Protein-Protein Interaction Networks (http://string-db.org)**


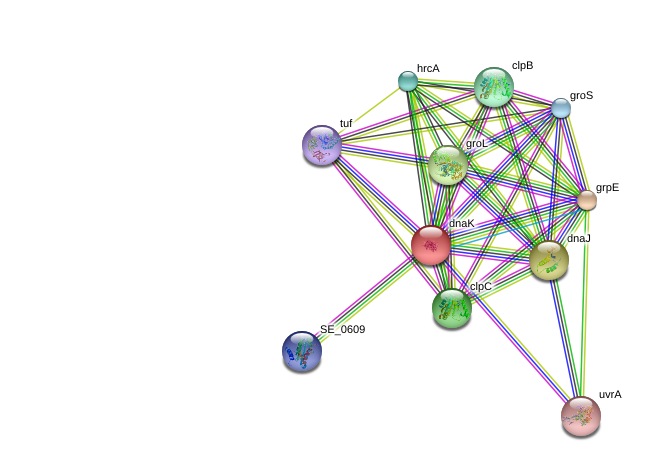
**Current Organism: *Staphylococcus epidermidis*** **ATCC 12228** NCBI taxonomy Id: 176280

|  | | | | | Neighborhood | Gene Fusion | Cooccurence | Coexpression | Experiments | Databases | Textmining | Homology | **Score** |
| --- | --- | --- | --- | --- | --- | --- | --- | --- | --- | --- | --- | --- | --- |
|  | 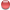 | dnaK | molecular chaperone DnaK (609 aa) |  |
| **Predicted Functional Partners** | | | | |
|  | 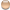 | grpE | heat shock protein GrpE (210 aa) | | [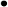](http://string-db.org/cgi/neighborhood.pl?taskId=XDAxBGArz9dE&node2=3402917) |  | [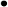](http://string-db.org/cgi/phylo_evidence.pl?taskId=XDAxBGArz9dE&node2=3402917) | [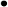](http://string-db.org/cgi/coexpression_evidence.pl?taskId=XDAxBGArz9dE&node2=3402917) | [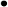](http://string-db.org/cgi/set_evidence.pl?data_channel=experimental&taskId=XDAxBGArz9dE&node2=3402917) | [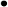](http://string-db.org/cgi/set_evidence.pl?data_channel=database&taskId=XDAxBGArz9dE&node2=3402917) | [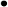](http://string-db.org/cgi/textmining.pl?taskId=XDAxBGArz9dE&node2=3402917) |  | 0.999 |
|  | 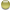 | dnaJ | molecular chaperone DnaJ (373 aa) | | [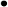](http://string-db.org/cgi/neighborhood.pl?taskId=XDAxBGArz9dE&node2=3402915) |  | [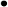](http://string-db.org/cgi/phylo_evidence.pl?taskId=XDAxBGArz9dE&node2=3402915) | [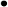](http://string-db.org/cgi/coexpression_evidence.pl?taskId=XDAxBGArz9dE&node2=3402915) | [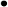](http://string-db.org/cgi/set_evidence.pl?data_channel=experimental&taskId=XDAxBGArz9dE&node2=3402915) |  | [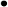](http://string-db.org/cgi/textmining.pl?taskId=XDAxBGArz9dE&node2=3402915) |  | 0.999 |
|  | 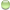 | groL | molecular chaperone GroEL (539 aa) | | [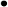](http://string-db.org/cgi/neighborhood.pl?taskId=XDAxBGArz9dE&node2=3403278) |  | [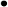](http://string-db.org/cgi/phylo_evidence.pl?taskId=XDAxBGArz9dE&node2=3403278) | [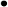](http://string-db.org/cgi/coexpression_evidence.pl?taskId=XDAxBGArz9dE&node2=3403278) | [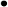](http://string-db.org/cgi/set_evidence.pl?data_channel=experimental&taskId=XDAxBGArz9dE&node2=3403278) |  | [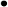](http://string-db.org/cgi/textmining.pl?taskId=XDAxBGArz9dE&node2=3403278) |  | 0.998 |
|  | 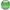 | clpC | endopeptidase (817 aa) | | [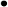](http://string-db.org/cgi/neighborhood.pl?taskId=XDAxBGArz9dE&node2=3401936) |  |  | [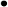](http://string-db.org/cgi/coexpression_evidence.pl?taskId=XDAxBGArz9dE&node2=3401936) | [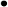](http://string-db.org/cgi/set_evidence.pl?data_channel=experimental&taskId=XDAxBGArz9dE&node2=3401936) |  | [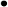](http://string-db.org/cgi/textmining.pl?taskId=XDAxBGArz9dE&node2=3401936) |  | 0.994 |
|  | 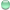 | clpB | clpB protein (869 aa) | | [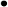](http://string-db.org/cgi/neighborhood.pl?taskId=XDAxBGArz9dE&node2=3402323) |  |  | [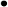](http://string-db.org/cgi/coexpression_evidence.pl?taskId=XDAxBGArz9dE&node2=3402323) | [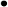](http://string-db.org/cgi/set_evidence.pl?data_channel=experimental&taskId=XDAxBGArz9dE&node2=3402323) |  | [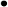](http://string-db.org/cgi/textmining.pl?taskId=XDAxBGArz9dE&node2=3402323) |  | 0.993 |
|  | 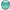 | hrcA | heat-inducible transcriptional repressor (325 aa) | | [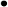](http://string-db.org/cgi/neighborhood.pl?taskId=XDAxBGArz9dE&node2=3402918) |  |  | [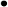](http://string-db.org/cgi/coexpression_evidence.pl?taskId=XDAxBGArz9dE&node2=3402918) |  |  | [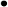](http://string-db.org/cgi/textmining.pl?taskId=XDAxBGArz9dE&node2=3402918) |  | 0.991 |
|  | 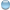 | groS | co-chaperonin GroES (94 aa) | | [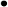](http://string-db.org/cgi/neighborhood.pl?taskId=XDAxBGArz9dE&node2=3403279) |  | [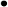](http://string-db.org/cgi/phylo_evidence.pl?taskId=XDAxBGArz9dE&node2=3403279) | [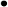](http://string-db.org/cgi/coexpression_evidence.pl?taskId=XDAxBGArz9dE&node2=3403279) | [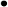](http://string-db.org/cgi/set_evidence.pl?data_channel=experimental&taskId=XDAxBGArz9dE&node2=3403279) |  | [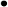](http://string-db.org/cgi/textmining.pl?taskId=XDAxBGArz9dE&node2=3403279) |  | 0.974 |
|  | 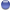 | SE_0609 | nitrogen fixation protein NifU (154 aa) | | [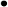](http://string-db.org/cgi/neighborhood.pl?taskId=XDAxBGArz9dE&node2=3402258) |  |  | [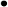](http://string-db.org/cgi/coexpression_evidence.pl?taskId=XDAxBGArz9dE&node2=3402258) | [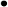](http://string-db.org/cgi/set_evidence.pl?data_channel=experimental&taskId=XDAxBGArz9dE&node2=3402258) |  | [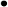](http://string-db.org/cgi/textmining.pl?taskId=XDAxBGArz9dE&node2=3402258) |  | 0.959 |
|  | 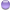 | tuf | elongation factor Tu (394 aa) | |  |  | [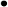](http://string-db.org/cgi/phylo_evidence.pl?taskId=XDAxBGArz9dE&node2=3401961) | [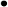](http://string-db.org/cgi/coexpression_evidence.pl?taskId=XDAxBGArz9dE&node2=3401961) | [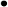](http://string-db.org/cgi/set_evidence.pl?data_channel=experimental&taskId=XDAxBGArz9dE&node2=3401961) |  | [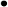](http://string-db.org/cgi/textmining.pl?taskId=XDAxBGArz9dE&node2=3401961) |  | 0.924 |
|  | 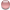 | uvrA | excinuclease ABC subunit A (944 aa) | |  |  | [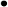](http://string-db.org/cgi/phylo_evidence.pl?taskId=XDAxBGArz9dE&node2=3402191) |  | [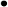](http://string-db.org/cgi/set_evidence.pl?data_channel=experimental&taskId=XDAxBGArz9dE&node2=3402191) |  | [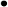](http://string-db.org/cgi/textmining.pl?taskId=XDAxBGArz9dE&node2=3402191) |  | 0.892 |

**Table S1. List of the 170 proteins identified in the secretome of control and CGRP-treated *S. epidermidis* by tandem mass spectrometry**
